# Multiscale Molecular Modeling in G Protein-Coupled Receptor (GPCR)-Ligand Studies

**DOI:** 10.3390/biom10040631

**Published:** 2020-04-19

**Authors:** Pratanphorn Nakliang, Raudah Lazim, Hyerim Chang, Sun Choi

**Affiliations:** College of Pharmacy and Graduate School of Pharmaceutical Sciences, Ewha Womans University, Seoul 03760, Korea; pnakliang@ewha.ac.kr (P.N.); raudah@ewha.ac.kr (R.L.); hrchang@ewhain.net (H.C.)

**Keywords:** G protein-coupled receptors (GPCRs), multiscale calculations, molecular modeling, structure-based drug design (SBDD)

## Abstract

G protein-coupled receptors (GPCRs) are major drug targets due to their ability to facilitate signal transduction across cell membranes, a process that is vital for many physiological functions to occur. The development of computational technology provides modern tools that permit accurate studies of the structures and properties of large chemical systems, such as enzymes and GPCRs, at the molecular level. The advent of multiscale molecular modeling permits the implementation of multiple levels of theories on a system of interest, for instance, assigning chemically relevant regions to high quantum mechanics (QM) level of theory while treating the rest of the system using classical force field (molecular mechanics (MM) potential). Multiscale QM/MM molecular modeling have far-reaching applications in the rational design of GPCR drugs/ligands by affording precise ligand binding configurations through the consideration of conformational plasticity. This enables the identification of key binding site residues that could be targeted to manipulate GPCR function. This review will focus on recent applications of multiscale QM/MM molecular simulations in GPCR studies that could boost the efficiency of future structure-based drug design (SBDD) strategies.

## 1. Introduction

G protein-coupled receptors (GPCRs) have been known as the largest family of human membrane protein that plays crucial roles in many biological processes such as vision, sensing, and neurotransmission [1,2,3,4,5,6,7,8]. Signal transmission through GPCRs is initiated by the binding of extracellular ligands including drugs, hormones, and other stimuli. The structural dynamics of GPCR as a consequence of ligand binding is considerably complex and contribute to its physiological functions. At the orthosteric binding site, GPCR ligands can be roughly divided into agonists and antagonists in which the first one activates the GPCR activity, whereas the latter act as blockers. Besides this, GPCR ligands can also bind to the allosteric site and indirectly affect the agonistic activity of GPCRs. Therefore, examining for ligand–receptor interactions that are vital in effectuating desirable GPCR functions has been the mainstream focus of most GPCR studies. To date, GPCRs are a target of more than 30% of approved drugs [9,10]. The understanding of the effects of ligands on GPCR properties has become the main task in rational drug and ligand designs. The importance of GPCR as viable drug targets was portrayed by the conferment of the Nobel Prize in Chemistry to Lefkowitz and Kobilka in 2012 for their groundbreaking discoveries on GPCRs [11]. The increase in structural data and the exploration of GPCR dynamics revealed the flexibility of its binding pockets as well as the tendency of GPCRs to adopt distinct conformations at different states. Therefore, computational simulation could serve as a complementary tool that could help in the discovery and design of GPCR ligands with desirable effects by permitting the scrutiny of GPCR dynamics.

Acquiring a comprehensive understanding of biomolecular drug interactions is essential in drug discovery. Many biological and biophysical techniques, such as X-ray crystallography, electron microscopy, cryo-electron microscopy (cryo-EM), small-angle X-ray scattering (SAXS), neutron scattering (SANS), and nuclear magnetic resonance spectroscopy (NMR), have been utilized to solve the three-dimensional conformations of proteins [12]. Currently, a large number of biological information has been published, providing resources required to probe proteins *in silico*, at the atomic level [5,6,13,14,15].

Molecular simulations provide the atomistic-level details useful for revealing and predicting important determinants influencing drug design such as binding affinity, reaction mechanism, and protein–ligand interactions. Molecular dynamics (MD) simulation, in particular, is becoming a powerful computational approach that applies classical molecular mechanics (MM) potential to simulate the time-dependent motions of atoms in the system of choice, hence providing researchers with the means to sample myriads of conformations that a protein may assume during biological processes at reasonable computational complexity. The MM method considers atoms and bonds as balls and springs, respectively. The physical properties and energy function of a system are calculated using force fields that are described by Newtonian mechanics. The cost-effectiveness of MD simulation saw its application to a wide variety of protein studies related to protein folding and unfolding, protein stability, structural transition, protein adaptation to different environments, and protein–ligand binding kinetics. However, an apparent setback of classical MM force field is its mean-field treatment of amino acid residues, hence rendering MD simulation unsuited for the elucidation of chemical reactions, which requires an accurate representation of the electronic structure of the system. This can be achieved via solving the Schrödinger equation in quantum mechanics (QM) calculation, a highly accurate computational approach that can be used for structure optimization, mechanistic studies of chemical reactions, and modeling of the optical properties of light-sensitive proteins, among many other uses. Contrary to MM force field, a limitation of the QM method is high computational demand and, therefore, is not appropriate for large systems (> hundreds of atoms) [12].

**Figure 1:**
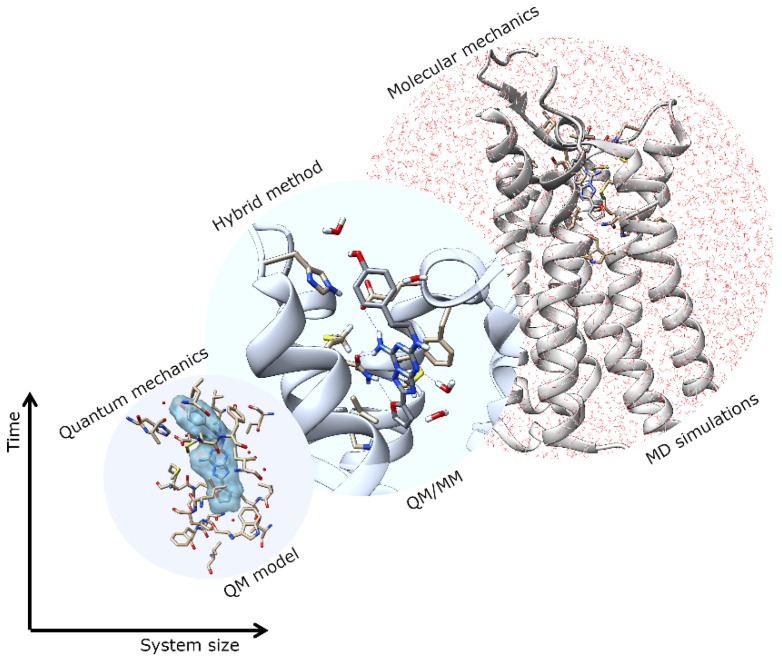
Multiscale modeling and application in computational simulations.

**Figure 2:**
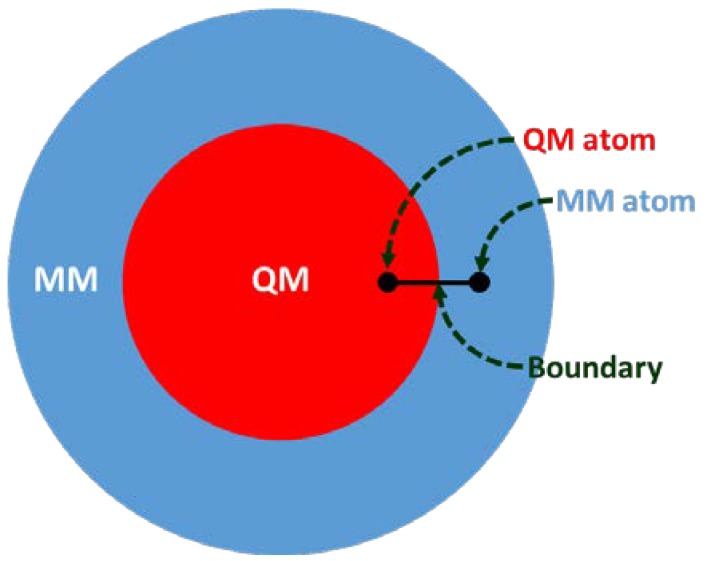
The hybrid quantum mechanics/ molecular mechanics (QM/MM) system partition and atoms at the QM-MM boundary.

Structure-based drug design (SBDD) is gaining tremendous attention for its potential in contributing to the design of novel drug candidates. With the continuous solving of the 3D conformations of GPCRs, a thorough understanding of the structural features of the receptors, such as orthosteric and allosteric binding site configurations, structural flexibility, and domain motions, can be attained and utilized in SBDD strategies for the development of novel GPCR drugs with ameliorated efficacy, efficiency, and specificity. The application of MD simulations in GPCR studies has been broadly reported, giving useful insights into ligand-binding interactions and GPCR dynamics [4,5,6,7]. However, to obtain a better picture of the binding site conformation as well as the chemical properties of ligands bound to GPCRs, more accurate descriptions of binding sites are necessary. Multiscale simulations are combinations of different time and length-scale resolution techniques (Figure 1). These techniques can be used at the same time or as a complementary method. The application of multiscale QM/MM simulation will be highly desirable for this purpose since this hybrid approach combines the advantages of both classical and QM theory, enabling the modeling of large complex chemical systems at a higher level of sophistication without exhausting too much computational resources [16,17,18]. In QM/MM molecular modeling, the enormous biological molecule will be divided into two (or more) regions, whereby a small region of interest (e.g., binding site) is described by the accurate QM method while the rest of system (protein, membrane, solvent, etc.) is treated using the less expensive MM level (Figure 2). Some examples of QM/MM applications in drug discovery include inhibitor design from transition state and/or intermediate structures, mechanism-based inhibitor design, prediction of drug metabolism, and observation of changes in the chemical nature of ligands due to protein conformational transitions. In 2013, the Nobel Prize in Chemistry was awarded to Karplus, Levitt, and Warshel for their contributions in developing multiscale molecular modeling for complex chemical systems, including biological molecules [19]. 

## 2. Molecular Docking Development using QM/MM Approach

The docking approach is a promising tool that is commonly utilized in SBDD efforts as a means to predict binding conformation of small molecules in the binding sites of target proteins. A number of docking software is available, for example, AutoDock [20], GOLD [21], GLIDE [22,23,24], and SwissDock [25], thus enabling facile determination of potential binding poses of ligands in proteins. The principle of docking simulations is based on the “lock-and-key” hypothesis, wherein the protein (host) is the lock while the ligand (guest) is the key, hence implying the specificity of a ligand to bind to a certain protein. During docking simulations, the orientation of guest(s) in the host is optimized and the accuracy of this process depends on two major components namely searching algorithm and scoring function [26]. Additionally, consideration of protein and ligand flexibility has been shown to play a crucial role in improving the accuracy of predicting ligand binding affinity to target protein. Thus, the efficiency of the docking method will be improved by including protein flexibility using induced-fit docking (IFD) or utilizing more than one host structure by acquiring a protein ensemble through MD simulations [27,28,29,30]. Recently, the QM/MM method was employed to improve the quality of docking simulations. The advantage of this approach is that the flexible protein environment is considered during the QM/MM calculation through simple geometry optimization of the assigned QM region in the presence of the free energy surface of the surrounding protein, which was constructed based on MM potential. The accuracy of the protocol was validated using a set of known protein–ligand structure complexes, which was taken from experimental data.

The implementation of QM/MM into docking protocol has been developed by many research groups to improve the accuracy for use in structure refinement and routine analysis involved in docking studies. The binding conformation of the ligand within the binding pocket of olfactory receptor MOR244-3 was studied [31]. The ligand-binding site contains a Cu(I) ion, which is responsible for the binding of the organosulfur odorant. In the study, the ligand–protein and ligand–Cu interactions are well characterized by the QM/MM description in which the QM region covers all important residues in the binding pocket. The calculated results are consistent with the mutagenesis studies of the receptor activation, which showed that the binding site consists of the Cu ion coordinating with His105, Cys109, and Asn202. Additional analyses performed using various ligands revealed that the thioether group is a significant part of the ligand-binding mechanism. The obtained results could be applied as a case study for other mammalian olfaction investigation. Recently, the activation of human odorant receptors, OR5AN1 and OR1A1, was studied to compare the calculated binding energies of (*R*)-muscone and other related compounds [32]. The theoretical results are in good agreement with the experimental results that indicate the preference for (*R*)- over (*S*)- enantiomer. Structural observation revealed that the ligand is stabilized by forming a hydrogen bond with Tyr260 and hydrophobic interactions with surrounding aromatic residues. This valuable finding may lead to the instructive development of the quantitative structure-activity relationship (QSAR) model. QM/MM simulation has also been utilized to improve the quality of the docking results of human dopamine D3 receptor (D3R), which has been identified as an antipsychotic drug target for schizophrenia treatment [33,34]. The well-known atypical antipsychotic (AAP) drugs include risperidone, aripiprazole, ziprasidone, clozapine, olanzapine, and quetiapine. All of these have been prescribed to treat various mental conditions [35]. The QM/MM minimization was performed on the selected docking poses. Only the ligand (haloperidol) was placed in the QM region, while the rest of the system was considered as the MM region. Accuracy of the interaction energy was shown to be dependent on the radius of the binding site that was included in the QM region during the calculation. It was due to the long-range interactions of distant charged residues that were included in the QM region. The interaction energy was calculated as −170.1 kcal/mol, which was larger than the other two classical methods used (−56.3 kcal/mol for classical mechanics minimization of all hydrogen atoms and haloperidol molecule, and −137.6 kcal/mol for only hydrogen atoms minimization). It indicated that the QM/MM refinement converged to the more stable conformation than classical minimization techniques. The combination of docking and QM/MM calculation revealed the important roles of surrounding amino acid residues in the binding pocket. Moreover, the hydroxyl group of haloperidol was identified as a major site that leads to stronger binding to dopamine receptors.

**Figure 3:**
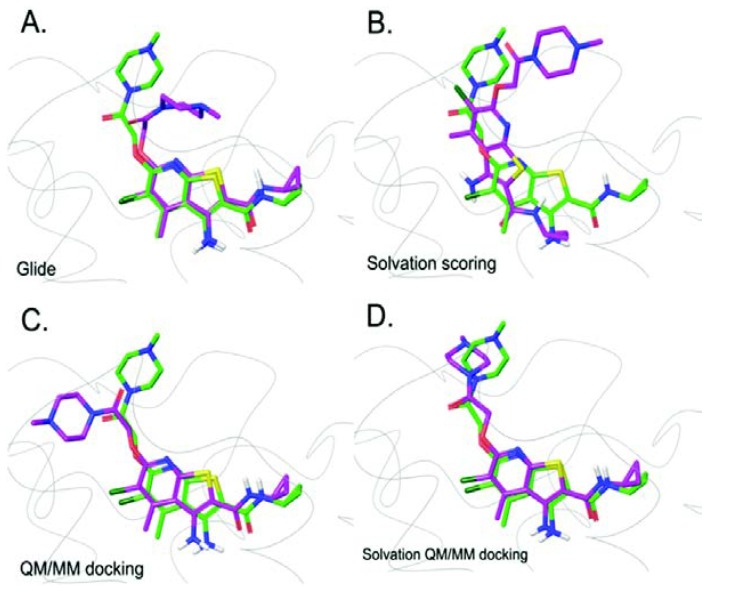
Prediction of LY2119620 binding modes using the four docking methods (green – crystal pose, purple – predicted pose). (**A**) Glide docking result. (**B**) Solvation scoring result. (**C**) QM/MM docking result. (**D**) Solvation QM/MM docking result. Heteroatoms are shown in yellow (S), red (O), blue (N), and dark green (Cl). Reproduced from Reference 43 with permission from the PCCP Owner Societies.

In 2005, the QM/MM simulation was incorporated into the docking algorithm, whereby the fixed charges of ligand assigned by MM force fields were replaced by partial charges fitted based on electrostatic potential of the ligand derived in the presence of protein environment during QM/MM calculation. Here, the ligand was the only molecule assigned as the QM region, while the rest of the system were described using the MM potential [36]. Cho et al. found that the use of polarized charges plays a significant role in improving the prediction of ligand binding mode and this leads to a new promising docking protocol for lead optimization in drug discovery. A subsequent study on metalloproteins suggested that the extension of the QM region to include metal ion(s) along with coordinated protein residues is important and leads to more reliable binding poses [37]. Due to the success of the incorporation of QM/MM into docking simulation, its applications in structure-based studies, including drug design, virtual screening, and lead optimization, have been investigated [38,39,40]. Current assessment of GPCR docking simulations without QM/MM showed that the success rate was over 70%. Docking error was evident especially for the docking of ZM241385 and XAC into Adenosine A_2A_ receptor [41,42]. In 2016, Kim and Cho incorporated QM and solvation effect into the docking simulation of GPCRs to improve the predicting accuracy. They proposed a new docking protocol that replaced the fixed force field charges of the ligand by partial charges calculated using QM/MM calculations with an extended QM region. This protocol was also used in re-docking simulations. The QM region used in the study included the ligand and surrounding amino acid residues within 5 Å of the ligand. The solvation effect was taken into account by solving the Poisson Boltzmann (PB) equation. Among a test set of 40 GPCR-ligand complexes, the QM/MM docking improved the success rate to 90% without solvation effect, which is better than the docking result from Glide with standard precision (Glide SP) and Glide with solvation effect included. The improvements in docking poses are shown in Figure 3. A possible issue of failed cases is the ligand containing solvent-exposed part(s). Therefore, integration of solvent effect into the QM/MM docking protocol by using an implicit solvent model was proposed. It demonstrated an excellent improvement with a success rate of 100%, portraying the importance of charge models in improving docking accuracy [43].

## 3. Class A Rhodopsin Photoactivity Investigation

Class A rhodopsin receptor is responsible for many physiological functions, particularly light-sensitive responses [6]. The biological activity of rhodopsin is initiated by light (photon energy). As a result of photoisomerization, it is possible to use rhodopsin as an energy storage material [44]. Therefore, photochemical events are the main topic of interest in most studies related to this receptor. The complete picture of photochemical reactions could be achieved computationally. A suitable computational method for rhodopsin photoactivity investigations is the QM/MM method that has been applied to understand structure, spectral tuning, photoisomerization, and mutations. Photoexcitation calculations demand high computational resources. Thus, retinal chromophore that is covalently bound to activated rhodopsin has been studied using many small models of the ligand to understand the rapid photoisomerization process [45,46]. Even though the gas phase calculated excitation energies are in good agreement with experiment, the effect of protein on the photochemical reaction was not explained using these models, especially the steric effects on the -ionone ring.

Methods aimed at understanding the effect of the protein environment on the photochemical process occurring during rhodopsin activation have been developed. The availability of high-resolution structural data accelerates the theoretical studies involving the structure–function relationship of rhodopsin. The photoisomerization of 11-*cis* rhodopsin to all-*trans* bathorhodopsin is one of the most attractive properties that has been widely investigated. A variety of hybrid methods have been used ranging from simple to complicated QM calculations. The energy difference between the minimum energies of rhodopsin and bathorhodopsin yielded energy storage of 34.1 kcal/mol, as calculated using QM/MM method at the B3LYP/6-31G*:AMBER level of theory. The result is in excellent agreement with experimental data [47,48,49]. The energy decomposition analysis revealed that large energy storage is due to the electronic interaction of rhodopsin. The rotation of the C11-C12 dihedral angle from −11° in 11-*cis* rhodopsin to −161° in all-*trans* bathorhodopsin was driven by the steric interaction between Ala117 and the polyene chain at the C13 position. This steric interaction hindered the rotation of the C11-C12 dihedral angle toward positive angles, an occurrence which could not be observed in the gas phase model. This study indicated that Glu113 may act as a counterion. Moreover, they suggested that the salt bridge between NH of the Schiff base linkage and Glu113 may be an important factor that influenced the electrostatic contribution of the protein to the total energy storage. The polarized bond at the Schiff linkage of bathorhodopsin shifted away from the negative site of Glu113 as compared to rhodopsin. The electrostatic contribution analysis of nearby residues in the binding pocket also provided insights on individual interactions, revealing that Ala117, Ser186, and a water molecule may stabilize bathorhodopsin relative to rhodopsin. The electronic-excitation energy estimation was also improved due to the integration of the electrostatic contribution of the protein environment during energy calculations.

With the increment in the number of available experimental GPCR structures, subsequent theoretical studies on rhodopsin have been in the spotlight [50,51,52,53]. In 2010, the structure and properties of squid rhodopsin were investigated. Similar to bovine rhodopsin, it contains 11-*cis* rhodopsin covalently bonded to Lys305. However, Glu113 in bovine rhodopsin is replaced by a group of Asn87, Tyr111, Glu180, and a water molecule. At that time, the position of internal water molecules could not be determined by X-ray crystallographic studies. Therefore, the number and positions of internal water molecules were verified by QM/MM calculation. It was found that the calculated structure of two additional water molecules near the Schiff base region is in good agreement with the X-ray structure. The absorption wavelength of retinal-chromophore blue-shifted around 120 nm when protein polarizability was accounted during the calculation. The effect of particular residues within 4 Å of the retinal polyene chain (34 amino residues) toward photoactivity of 11-*cis* rhodopsin was calculated by turning off the charges of these residues, one at a time. Among these residues, Glu180 blue-shifted the absorption wavelength by around 100 nm and was identified as the main counterion in squid rhodopsin. They suggested that even though Glu180 is located further away from the retinal chromophore compared to Glu113 in bovine rhodopsin, the charge stabilization engendered by Glu180 still has a significant effect on the optical properties of squid rhodopsin.

The QM/MM calculation of Class A rhodopsin GPCRs also provided a new perspective on *retinitis pigmentosa*, a disease involving progressive retinal degradation [54,55]. Rhodopsin mutations have been identified as a major cause of this disease [56]. Therefore, many mutagenesis studies have been conducted to determine key residues that may contribute to the development of *retinitis pigmentosa*. However, the mechanisms and causes of mutation are not clear. Hernández-Rodríguez et al. studied two mutated human rhodopsins (S186W and M207R) and compared the mutated models to that of wild type. The protein models were solvated in water and phosphatidylcholine (POPC) lipid bilayer. A combination of various computational methods, namely MD simulations, density functional theory (DFT), and QM/MM, was applied. The results unveiled that a less stable counterion region could impair the whole protein in the mutated models. Moreover, the strong blue-shift resulting from the mutations leads to excess energy that could yield side reactions. The results of this study could be utilized to support the rational development of medical treatment.

Besides the effect of protein environment, the structure of the retinal ligand itself also plays an important role in photoisomerization. The *cis-trans* isomerization of rhodopsin and isorhodopsin was studied using a combination of QM/MM and MD simulations [57]. Isorhodopsin is a rhodopsin analog that has a 9-*cis* retinal chromophore instead of an 11-*cis* retinal chromophore. MD simulations suggested that isomerization is a fast and facile event in rhodopsin, while being a much more complicated phenomenon in isorhodopsin. The 9-*cis* position in the retinal ligand of isorhodopsin forms a steric hindrance within the narrow space inside the opsin, thus affording byproducts. QM/MM calculations simulating the photoactivity of both systems showed that isorhodopsin photoisomerization gave rise to alternative products such as the 9,11-di-cis isomer. This is in contrary to the straightforward bathorhodopsin-only pathway in rhodopsin isomerization. Therefore, rhodopsin is preferred in nature. According to the simulations, protein environments, counterion, and chromophore structures are key factors that governed the photoactivity of rhodopsin. Incorporation of QM/MM simulations would broaden the understanding of particular state of rhodopsin photoactivation related diseases. The obtained knowledge can be utilized in drug design that target to stabilize the degradation of rhodopsin [58].

Currently, many complicated simulations are accessible. As mentioned above, the function of rhodopsin depends on many factors and understanding the protein–ligand interactions of rhodopsin is vital for the rational design of novel ligands and biomimicking molecules. The automatic rhodopsin modeling (ARM) method was proposed to study and predict the optical properties of class A rhodopsin system [59]. The protocol of this theoretical tool is as follows; (i) chromophore cavity definition, (ii) protonation state of amino acid residues, (iii) counterion position, and (iv) appropriate generation of mutation residue(s) for further parallel studies. Based on their benchmark test set, the computational maximum absorption wavelength ($\lambda_{max}^{a}$) showed excellent agreement with observed experimental data. As a result, automatic a-ARM provides high reproducibility (user-independent). Moreover, the utilization of ARM reduced the preparation time and also provided a practical simulation protocol for rhodopsin and other classes of GPCRs. The detailed structure–function and energetic analysis will provide a complete picture of the class A rhodopsin and also mutation-specific therapies.

## 4. The QM Approach in GPCR Studies

**Figure 4:**
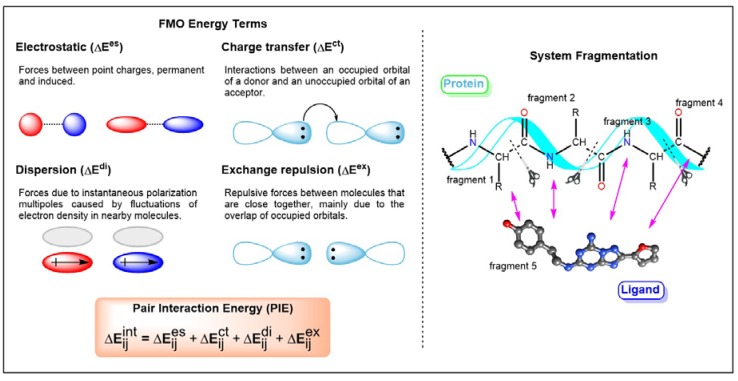
Workflow of the fragment molecular orbital (FMO) method and details of pair interaction energy (PIE) terms. Adapted from Reference 64 with permission.

Recently, an approximate molecular orbital (MO) method called Fragment Molecular Orbital (FMO) was implemented into studies related to GPCR-ligand interactions [60,61,62,63,64,65,66,67,68]. FMO has been described in previous publications and review articles [60,61,62]. Therefore, only a brief introduction of this method is presented here. The *modus operandi* of FMO involves the division of the system into fragments followed by QM calculations of each fragment. This method reduces the amount of time required to conduct QM calculations of the whole system. The interaction between two fragments is characterized by electrostatics, exchange-repulsion, charge transfer, and dispersion interactions (Figure 4). Therefore, the application of FMO on GPCR-ligand studies would yield reliable protein–ligand interactions that are important for biomolecular recognition. The information obtained is useful for SBDD. Weak interactions such as halogen bonds, cation-interactions, and non-classical hydrogen bonds that could not be explained by the MM force field could be achieved through the FMO method. These interactions have been shown to be key features in biological processes such as ligand recognition and protein folding. The theoretical characterization of ligand-binding recognition in GPCRs exhibited similar electrostatic and hydrophobic interactions across most GPCR complexes. In 2016, Heifetz and coworkers performed the FMO calculation on the complexes of agonist-orexin-2 receptor (OX_2_R) [64]. They considered all interactions with an absolute pair interaction energy (PIE) greater than or equal to 3.0 kcal/mol. A comparison of the interactions of two docking poses indicated that they shared similar interactions, and this was supported by site-directed mutagenesis studies. Subsequently, GPCR-ligand crystal structures were investigated [65]. They revealed the often omitted interactions contributed by surrounding residues, especially hydrophobic interaction and the involvement of backbone atoms. Comprehensive QM studies of protein–ligand interactions provide valuable information for rational SBDD. For instance, which ligand fragments could be targeted for modification to achieve desired properties [68]. Data on protein–ligand interactions acquired based on the FMO method have been published online (https://drugdesign.riken.jp/FMODB/) [69]. Currently, more than 980 unique PDB entries were identified. Moreover, an automated FMO calculation protocol was also developed in 2019 [70]. It is a valuable guideline for mutagenesis, interaction studies, and protein engineering.

## 5. Conclusions and Outlooks

GPCRs are important membrane proteins that play key roles in numerous physiological processes. GPCR–ligand (drug) interactions are crucial in modulating GPCR activity. Thus, a detailed understanding of GPCR–ligand interaction is needed for the design and development of new GPCR therapeutics. Previously, most of SBDD efforts in GPCR studies employed classical molecular docking that allows researchers to achieve their goals effectively. However, the development of multiscale molecular modeling has resulted in the reduction of computational demand for a relatively high-level accuracy approach such as QM. The application of a sophisticated computational strategy to a large and complex GPCR system was made feasible through QM/MM method, thus providing a practical prediction method that offers new insights into the structure, interaction, dynamics, and kinetics of GPCRs. Furthermore, the incorporation of QM in calculations provides missing pieces of important weak protein–ligand interactions such as hydrogen bond, cation-π, and non-classical hydrogen bond, which could not be determined by classical MM methods. Thus, it will improve the current SBDD protocol, making it valuable for pharmaceutical research in the near future.
